# Additively Manufactured Zirconia for Dental Applications

**DOI:** 10.3390/ma14133694

**Published:** 2021-07-01

**Authors:** Hiroto Nakai, Masanao Inokoshi, Kosuke Nozaki, Keiji Komatsu, Shingo Kamijo, Hengyi Liu, Makoto Shimizubata, Shunsuke Minakuchi, Bart Van Meerbeek, Jef Vleugels, Fei Zhang

**Affiliations:** 1 Department of Gerodontology and Oral Rehabilitation, Graduate School of Medical and Dental Sciences, Tokyo Medical and Dental University, Tokyo 113-8549, Japan; hnakgerd@tmd.ac.jp (H.N.); liugerd@tmd.ac.jp (H.L.); makotobata@gmail.com (M.S.); s.minakuchi.gerd@tmd.ac.jp (S.M.); 2 Department of Fixed Prosthodontics, Graduate School of Medical and Dental Sciences, Tokyo Medical and Dental University, Tokyo 113-8549, Japan; k.nozaki.fpro@tmd.ac.jp; 3 Department of Materials Science and Technology, Nagaoka University of Technology, Nagaoka 940-2188, Japan; kkomatsu@vos.nagaokaut.ac.jp; 4 Basic Oral Health Engineering, Graduate School of Medical and Dental Sciences, Tokyo Medical and Dental University, Tokyo 113-8549, Japan; s-kamijoh.itoe@tmd.ac.jp; 5 Department of Oral Health Sciences, BIOMAT & UZ Leuven (University Hospitals Leuven), Dentistry, KU Leuven (University of Leuven), 3000 Leuven, Belgium; bart.vanmeerbeek@kuleuven.be (B.V.M.); fei.zhang@kuleuven.be (F.Z.); 6 Department of Materials Engineering, KU Leuven (University of Leuven), 3001 Leuven, Belgium; jozef.vleugels@kuleuven.be

**Keywords:** zirconia, additive manufacturing, subtractive manufacturing, alumina-toughened zirconia, X-ray diffraction (XRD), Rietveld refinement, Weibull analysis, microstructural analysis

## Abstract

We aimed to assess the crystallography, microstructure and flexural strength of zirconia-based ceramics made by stereolithography (SLA). Two additively manufactured 3 mol% yttria-stabilized tetragonal zirconia polycrystals (3Y-TZP: LithaCon 3Y 230, Lithoz; 3D Mix zirconia, 3DCeram Sinto) and one alumina-toughened zirconia (ATZ: 3D Mix ATZ, 3DCeram Sinto) were compared to subtractively manufactured 3Y-TZP (control: LAVA Plus, 3M Oral Care). Crystallographic analysis was conducted by X-ray diffraction. Top surfaces and cross-sections of the subsurface microstructure were characterized using scanning electron microscopy (SEM). Biaxial flexural strength was statistically compared using Weibull analysis. The additively and subtractively manufactured zirconia grades revealed a similar phase composition. The residual porosity of the SLA 3Y-TZPs and ATZ was comparable to that of subtractively manufactured 3Y-TZP. Weibull analysis revealed that the additively manufactured LithaCon 3Y 230 (Lithoz) had a significantly lower biaxial flexural strength than 3D Mix ATZ (3D Ceram Sinto). The biaxial flexural strength of the subtractively manufactured LAVA Plus (3M Oral Care) was in between those of the additively manufactured 3Y-TZPs, with the additively manufactured ATZ significantly outperforming the subtractively manufactured 3Y-TZP. Additively manufactured 3Y-TZP showed comparable crystallography, microstructure and flexural strength as the subtractively manufactured zirconia, thus potentially being a good option for dental implants.

## 1. Introduction

Zirconia ceramic has widely been employed in dentistry as alternative of metal for dental restorations, due to its excellent biocompatibility, mechanical properties, and aesthetics compared to metal. More recently, zirconia ceramic has been used to fabricate dental implants [1,2].

Most of the zirconia-based restorations are fabricated using subtractive manufacturing methods, such as machining and milling [3]. Along with the development of digital dentistry, additive manufacturing is attractive with a high potential of making customized dental prostheses at minimal waste. Among different techniques, few methodologies can be used to fabricate fully dense ceramic, with stereolithography being one of the most promising techniques [4,5,6,7,8]. It has also been shown that zirconia ceramic can be fabricated with sufficient accuracy and strength [9,10]. Current commercially available equipment makes use of two different technologies, being direct light processing (DLP) and laser-based stereolithography (SLA) [11]. Applying additive manufacturing methods to fabricate dental implants can be advantageous as they can directly form complex topographies during fabrication [12], which improves the osteoinductive activity without any surface damage created by surface treatments, such as sandblasting. Usually, zirconia implants are processed by hard machining from a sintered cylinder, or by injection molding or mold filling of zirconia powder and cold isostatic pressing, followed by de-binding and sintering [13]. The machined or sintered implants are sandblasted and acid-etched to obtain a sufficiently rough surface for osteoinductivity. However, sandblasting may induce micro-cracks and may affect mechanical properties [14,15]. With additive manufacturing, sandblasting or acid etching can be avoided, which is beneficial for the strength and reliability of zirconia implants.

Thus far, some studies have focused on additive manufacturing methods to fabricate zirconia-ceramic implants using commercially available systems. However, the results are inconclusive [16,17]. Osman et al. (2017) assessed the dimensional accuracy and surface topography of additively manufactured zirconia dental implants and the mechanical properties of additively manufactured zirconia disks [16]. They reported sufficient dimensional accuracy and comparable flexural strength of additively and subtractively manufactured zirconia. On the other hand, Revilla-León et al. (2021) reported a lower flexural strength for additively than for subtractively manufactured zirconia [17]. A systematic comparison between subtractively and additively manufactured zirconia is needed to clarify the potential of additively manufactured zirconia for dental implant fabrication.

Not only 3 mol% yttria-stabilized tetragonal zirconia polycrystals (3Y-TZP), but also alumina-toughened zirconia (ATZ) is interesting as titanium alternative to fabricate dental implants [18,19]. This is because ATZ has a higher flexural strength with better aging resistance compared to 3Y-TZP [20,21].

This study aimed to assess the crystal structure, morphology and mechanical properties of additively manufactured zirconia-based ceramics in comparison to subtractively manufactured zirconia. The null hypothesis tested was that the crystal structure, microstructure and mechanical properties of additively manufactured zirconia-based ceramics are comparable to those of subtractively manufactured zirconia. 

## 2. Materials and Methods

**Figure 1:**
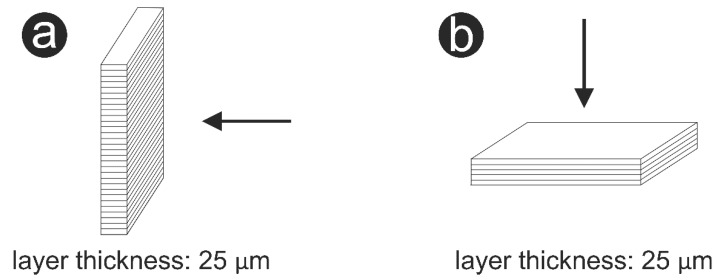
Schematic showing the building direction of the additively manufactured specimens. (**a**): 90°; (**b**): 0°. Arrows indicate the load direction during the biaxial flexural strength test.

A summary of the characteristics and properties of the ceramics investigated is provided in Table 1, including two additively manufactured 3Y-TZPs (LithaCon 3Y 230, Lithoz, Vienna, Austria; 3D Mix zirconia, 3DCeram Sinto, Limoges, France), one additively manufactured ATZ (3D Mix ATZ, 3DCeram Sinto, Limoges, France) and one subtractive manufactured zirconia (LAVA Plus, 3M Oral Care, Seefeld, Germany). All specimens were prepared by the manufacturers in square shape with a dimension of 12 mm × 12 mm × 1.2 mm. The building direction of the specimens was 90° for LithaCon 3Y 230 (Lithoz) and 0° for 3D Mix zirconia and 3D Mix ATZ (3DCeram Sinto), as shown in Figure 1.

### 2.1. Crystal Structure

X-ray diffraction (XRD, D8 Advance, Bruker, Ettlingen, Germany) with Cu K_α_ (40 kV, 40 mA) was employed for phase identification and calculation of the relative phase content of cubic zirconia (*c*-ZrO_2_), tetragonal zirconia (*t*-ZrO_2_), monoclinic zirconia (*m*-ZrO_2_) and alumina (Al_2_O_3_). Rietveld analysis was used to assess the zirconia and ATZ phase composition using TOPAS academic V7 software (Coelho software, Brisbane, Australia).

### 2.2. Flexural Strength

The flexural strength was determined using a biaxial flexural strength test (n = 14–15/group) with a piston on three-ball set-up following a method for square-shaped specimens introduced by Cokic et al. and Wendler et al. [22,23]. The specimens were loaded at a crosshead speed of 0.5 mm/min until failure in a universal testing machine (EZ-LX, Shimadzu, Tokyo, Japan). The Poisson ratio of the zirconia grades was set as 0.3, whereas that of ATZ was set to 0.27, following previous studies [14,24]. The flexural strength results were statistically analyzed using Weibull analysis. Weibull parameters were calculated by maximum-likelihood estimation. The likelihood ratio was used to calculate the confidence interval bounds. Moreover, a likelihood contour method was employed to determine the statistical difference of the compared Weibull distributions [25]. All tests were performed at a significance level of α = 0.05 using a software package R3.6.1 and weibullR (R Foundation for Statistical Computing, Vienna, Austria).

### 2.3. Microstructural Analysis

The top surfaces of the specimens from each experimental group were investigated for microstructural analysis using scanning electron microscopy (SEM: S-4500 Hitachi, Tokyo, Japan). The specimens were coated with a thin layer of Pt (E102 Ion Sputter, Hitachi, Tokyo, Japan). The surfaces were investigated at an accelerating voltage of 5 kV, emission current of 8 μA and working distance of 10 mm. In addition, one of the fractured specimens from each experimental group was cross-sectioned, polished and argon-ion milled (Cross Section Polisher, SM-09010; JEOL, Tokyo, Japan). A thin layer of Pt was coated on the samples prior to examination in backscattered electron imaging mode using a field-emission-gun SEM (FE-SEM; Hitachi SU8230, Hitachi) operated at 15 kV. The elemental distributions for the samples were determined using energy dispersive X-ray spectroscopy (EDS).

## 3. Results

### 3.1. Crystal Structure

**Figure 2:**
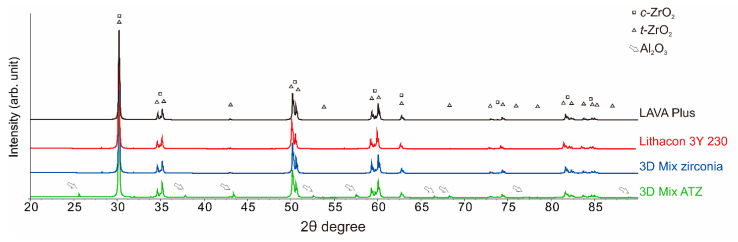
Representative X-ray diffraction (XRD) patterns for the 3Y-TZPs and ATZ investigated. The subtractively manufactured zirconia (LAVA Plus, 3M Oral Care) and additively manufactured zirconia (LithaCon 3Y 230, Lythoz; 3D Mix zirconia, 3DCeram Sinto) present comparable XRD patterns. On the other hand, 3D Mix ATZ (3DCeram Sinto) clearly shows Al_2_O_3_ peaks (open white arrows).

Regarding the phase composition of the investigated zirconia(-based) ceramics, the results of the Rietveld analysis are detailed in Table 2. Representative XRD patterns are shown in Figure 2. XRD with Rietveld analysis revealed that additively manufactured zirconia (LithaCon 3Y 230, Lithoz; 3D Mix zirconia, 3DCeram Sinto) and subtractively manufactured zirconia (LAVA Plus, 3M Oral Care) contained 86–88 wt% of *t*-ZrO_2_ phase, being typical 3Y-TZP ceramics, whereas additively manufactured ATZ (3D Mix ATZ, 3DCeram Sinto) contained approximately 20 wt% of Al_2_O_3_ phase.

### 3.2. Flexural Strength

**Figure 3:**
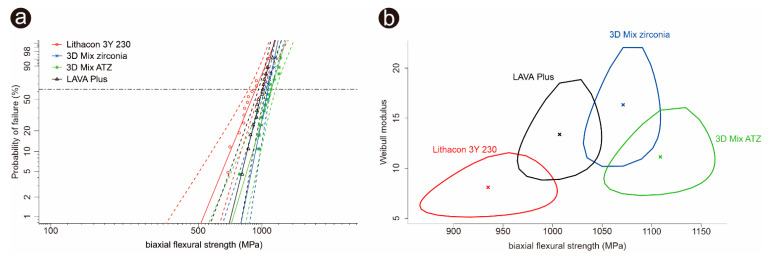
Summary of the Weibull analysis. (**a**): Weibull plots for the 3Y-TZPs and ATZ investigated. (**b**): Weibull contour plots (95% confidence intervals) for 3Y-TZPs and ATZ. Weibull modulus and characteristic strength of each ceramic grade are presented with “×” inside of the contour plots.

Figure 3 and Table 3 summarize the biaxial flexural strength results from the Weibull analysis. Weibull analysis revealed that among the two additively manufactured zirconia (LithaCon 3Y 230, Lithoz; 3D Mix zirconia, 3DCeram Sinto), 3D Mix zirconia had a significantly higher biaxial flexural strength than LithaCon 3Y 230 (Lithoz). The biaxial flexural strength of the additively manufactured 3Y-TZPs (3D Mix zirconia, 3DCeram Sinto) was comparable to that of additively manufactured ATZ (3D Mix ATZ, 3DCeram Sinto), which is higher than that of subtractively manufactured 3Y-TZP (LAVA Plus, 3M Oral Care). The biaxial strength of additively manufactured LithaCon 3Y 230 (Lithoz) was the lowest.

### 3.3. Microstructural Analysis

**Figure 4:**
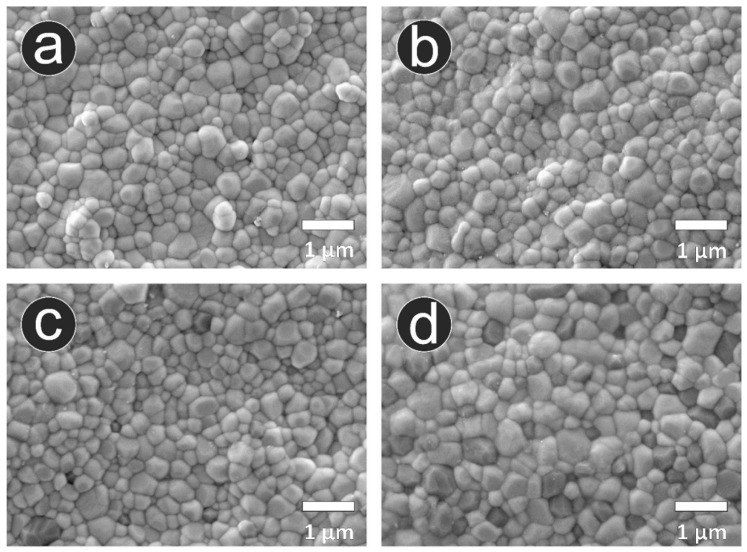
Representative SEM photomicrographs. (**a**) subtractively manufactured 3Y-TZP (LAVA Plus, 3M Oral care); (**b**) additively manufactured 3Y-TZP (LithaCon 3Y 230, Lithoz); (**c**) additively manufactured 3Y-TZP (3D Mix zirconia, 3DCeram Sinto); (**d**) additively manufactured ATZ (3D Mix ATZ, 3DCeram Sinto).

**Figure 5:**
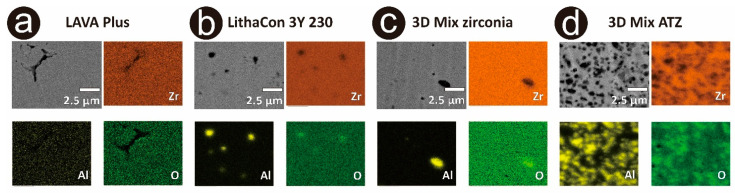
Representative FE-SEM photomicrographs and EDS maps of Zr, Al, and O. (**a**) subtractively manufactured 3Y-TZP (LAVA Plus, 3M Oral Care); (**b**) additively manufactured 3Y-TZP (LithaCon 3Y 230, Lithoz); (**c**) additively manufactured 3Y-TZP (3D Mix zirconia, 3DCeram Sinto); (**d**) additively manufactured zirconia (3D Mix ATZ, 3DCeram Sinto).

Representative SEM images are presented in Figure 4. Secondary as well as backscattered electron images and results of the EDS elemental analysis are shown in Figure 5. Microstructural analysis revealed a comparable microstructure for the additively manufactured 3Y-TZPs (LithaCon 3Y 230, Lithoz; 3D Mix zirconia, 3DCeram Sinto) and subtractively manufactured 3Y-TZP (LAVA Plus, 3M Oral Care). For the additively manufactured ATZ (3D Mix ATZ, 3DCeram Sinto), a high amount of darker contrast alumina grains was observed. The additively manufactured 3Y-TZP (LithaCon 3Y 230, Lithoz; 3D Mix zirconia, 3DCeram Sinto) specimens show comparable microstructural photomicrographs. Imaging cross-sectioned samples, the subtractively manufactured 3Y-TZP (LAVA Plus, 3M Oral Care: Figure 5a) showed a lower amount of Al_2_O_3_ than the additively manufactured 3Y-TZPs (LithaCon 3Y 230, Lithoz: Figure 5b; 3D Mix zirconia, 3DCeram Sinto: Figure 5c). Moreover, pores were more frequently detected on subtractively manufactured 3Y-TZP (LAVA Plus, 3M Oral Care) than additively manufactured 3Y-TZP (LithaCon 3Y 230, Lithoz; 3D Mix zirconia, 3DCeram Sinto). For the cross-sectioned additively manufactured zirconia, the Al_2_O_3_ content in LithaCon 3Y 230 (Lithoz) and 3D Mix zirconia (3DCeram Sinto) were comparable, whereas the cross-sectioned additively manufactured ATZ (3D Mix ATZ, 3DCeram Sinto) had a substantially higher amount of Al_2_O_3_. 

## 4. Discussion

The present study investigated the crystal structure, microstructure, and mechanical properties of additively manufactured 3Y-TZP and ATZ in comparison with conventionally fabricated 3Y-TZP. XRD with Rietveld analysis revealed that additively manufactured 3Y-TZP (LithaCon 3Y 230, Lithoz; 3D Mix zirconia, 3DCeram Sinto) and subtractively manufactured 3Y-TZP (LAVA Plus, 3M Oral Care) showed comparable phase composition. Regarding biaxial flexural strength, significant differences were observed between subtractively manufactured 3Y-TZP (LAVA plus, 3M Oral Care) and additively manufactured ATZ (3D Mix ATZ, 3DCeram Sinto). Microstructural analysis revealed that pores were more frequently detected in subtractively manufactured zirconia (LAVA Plus, 3M Oral Care) than in additively manufactured 3Y-TZPs (LithaCon 3Y 230, Lithoz; 3D Mix zirconia, 3DCeram Sinto) and ATZ (3D Mix ATZ, 3DCeram Sinto). Therefore, the null hypothesis that the crystal structure, microstructure and mechanical properties of additively manufactured zirconia ceramics and alumina toughened zirconia are comparable to subtractively manufactured zirconia has been partially rejected.

According to the XRD phase analysis, the present study demonstrated that additively manufactured zirconia ceramics (LithaCon 3Y 230, Lithoz; 3D Mix zirconia, 3DCeram Sinto) have a comparable phase composition as subtractively manufactured zirconia (LAVA Plus, 3M Oral Care). Osman et al. (2017) assessed the zirconia-phase composition using XRD and showed that additively manufactured zirconia ceramics had similar XRD patterns to 3Y-TZP [16]. Zhang et al. (2017) presented similar XRD patterns for both additively and subtractively manufactured zirconia [26]. Our results are in line with these studies, and the zirconia-phase composition is determined by the yttria content of the zirconia powder in the printing slurry.

Biaxial flexural strength testing revealed that additively manufactured 3Y-TZP can have a strength similar to that of subtractively manufactured 3Y-TZP. Even better, this study demonstrated the possibility of manufacturing zirconia ceramics with better reliability by SLA technology, as 3D Mix zirconia (3DCeram Sinto) showed a higher Weibull modulus than LAVA plus (3M Oral Care). This could be partially due to the fact that SLA is based on a slurry instead of dry powder. However, among the additively manufactured 3Y-TZPs, LithaCon 3Y 230 (Lithoz) had a significantly lower biaxial flexural strength than 3D Mix zirconia (3DCeram Sinto). This can be related to differences in their manufacturing method: LithaCon 3Y 230 (Lithoz) was fabricated by digital light printing (DLP)-stereolithography (SLA), whereas 3D Mix zirconia (3DCeram Sinto) was fabricated by laser-based SLA. The building direction affected the biaxial flexural strength of the additively manufactured 3Y-TZPs as well. In the present study, the building direction of LithaCon 3Y 230 (Lithoz) and 3D Mix zirconia (3DCeram Sinto) was different: LithaCon 3Y 230 (Lithoz) was printed horizontally to the load direction, whereas 3D Mix zirconia (3DCeram Sinto) was printed vertically to the load direction. In general, specimens in which the building and tensile loading direction are parallel (Figure 1a) have an inferior flexural strength than specimens which are loaded perpendicularly to the building direction (Figure 1b) [9]. Our results were in line with a previous study reported by Bergler et al. (2021), which showed comparable flexural strength for both additively and subtractively manufactured zirconia [27]. On the other hand, Lu et al. and Revilla-Leon et al. reported that additive manufactured zirconia had a lower flexural strength than subtractive manufactured zirconia [17,28]. Further investigations are needed to investigate the influence of anisotropy on the mechanical properties of additively manufactured zirconia.

As previously reported, ATZ is stronger than 3Y-TZP [21,29]. This is in line with our results. Moreover, the biaxial flexural strength of additively manufactured ATZ in the present study was comparable to that of subtractively manufactured ATZ tested in a previous study [30]. Microstructural analysis revealed that more pores were detected in subtractively than additively manufactured zirconia. In the case of zirconia implants, pores created during fabrication may affect their mechanical properties [12]. Using SEM, pores and alumina are difficult to be clearly distinguished, as they both have a dark contrast. Therefore, pores were detected by EDS elemental mapping [31]. We expected that additively manufactured zirconia would have more residual porosity because of their layer-by-layer fabrication method. Apparently, the well-controlled additive manufacturing method does not increase porosity during fabrication. Moreover, EDS revealed that the subtractively manufactured zirconia (LAVA Plus, 3M Oral Care) had a lower Al_2_O_3_ content than the additively manufactured 3Y-TZPs (LithaCon 3Y 230, Lithoz; 3D Mix zirconia, 3DCeram Sinto), which is in line with their overall composition. LAVA Plus (3M Oral Care) is categorized as a highly translucent 3Y-TZP (0.05 wt% Al_2_O_3_) that contains less Al_2_O_3_ than a conventional 3Y-TZP (0.25 wt% Al_2_O_3_) [32].

In the present study, we only investigated two additively manufactured zirconia grades. As mentioned above, the influence of the building direction on the mechanical properties of additively manufactured zirconia should be investigated. Moreover, fatigue resistance of additively manufactured zirconia could be an important property to tackle in the future.

We believe that additively manufactured 3Y-TZP and ATZ could be suitable for dental implants, since additive manufacturing can also realize a complex surface topography during fabrication. Moreover, the influence of surface morphology on the osteoinductive activity of additively manufactured zirconia remains to be investigated.

## 5. Conclusions

Additively manufactured zirconia revealed a crystal structure, biaxial flexural strength and microstructure comparable to that of subtractively (conventionally) manufactured zirconia. Differences in the additive manufacturing process of zirconia may affect the biaxial flexural strength of additively manufactured zirconia. Additively manufactured ATZ had a higher biaxial flexural strength than additively and subtractively manufactured 3Y-TZP.

## Figures and Tables

**Table 1:** Details of the zirconia ceramics investigated.

Zirconia Grades	Manufacturer	Zirconia Kind	Manufacturing	Lot
LAVA Plus	3M Oral Care	3Y-TZP	Subtractive	6433168
LithaCon 3Y 230	Lithoz	3Y-TZP	Additive	-
3D Mix zirconia	3DCeram Sinto	3Y-TZP	Additive	ZRJ 004-019
3D Mix ATZ	3DCeram Sinto	ATZ	Additive	ATZ-F01060720

**Table 2:** Relative amounts of each phase for the 3Y-TZPs and ATZ investigated.

Zirconia Grades	Phase Composition (wt%)				Goodness of Fit
	*t*-ZrO_2_	*c*-ZrO_2_	*m*-ZrO_2_	Al_2_O_3_	
LAVA Plus	86.4	13.4	0.1	-	1.19
LithaCon 3Y 230	87.6	12.2	0.2	-	1.35
3D Mix zirconia	88.5	11.2	0.3	-	1.20
3D Mix ATZ	68.3	11.0	0.3	20.4	1.22

**Table 3:** Summary of the Weibull biaxial strength analysis.

Zirconia Grades	Shape (Modulus)	95% Confidence Level at Modulus	Scale (B63.2)	95% Confidence Level at B63.2
LAVA Plus	13.4	8.3–18.3	1007.0	964.4–1049.5 (bc)
LithaCon 3Y 230	8.1	4.8–11.2	934.8	865.8–1004.7 (c)
3D Mix zirconia	16.3	9.6–21.4	1071.1	1031.0–1109.0 (ab)
3D Mix ATZ	11.1	6.8–15.6	1108.8	1051.7–1163.9 (a)

## Data Availability

The data presented in this study are available from the corresponding author, M.I., upon reasonable request.
